# Using NMR to Dissect the Chemical Space and *O*-Sulfation Effects within the *O*- and *S*-Glycoside Analogues of Heparan Sulfate

**DOI:** 10.1021/acsomega.2c02070

**Published:** 2022-07-08

**Authors:** Maria C.Z. Meneghetti, Lucy Naughton, Conor O’Shea, Dindet S.-E. Koffi Teki, Vincent Chagnault, Helena B. Nader, Timothy R. Rudd, Edwin A. Yates, José Kovensky, Gavin J. Miller, Marcelo A. Lima

**Affiliations:** † Departamento de Bioquímica, Instituto de Farmacologia e Biologia Molecular, Escola Paulista de Medicina, Universidade Federal de São Paulo, Rua Três de Maio, 100, São Paulo 04044-020, São Paulo, Brazil; ‡ School of Life Sciences, Keele University, Keele ST55BG, Staffordshire, U.K.; § Centre for Glycosciences, Keele University, Keele ST55BG, Staffordshire, U.K.; ∥ Lennard-Jones Laboratories, School of Chemical and Physical Sciences, Keele University, Keele ST55BG, Staffordshire, U.K.; ⊥ Laboratoire de Glycochimie, des Antimicrobiens et des Agroressources (LG2A), UMR 7378 CNRS, Université de Picardie Jules Verne, 33 rue Saint Leu, Amiens Cedex F-80039, France; # National Institute for Biological Standards and Control (NIBSC), Blanche Lane, South Mimms, Potters Bar EN6 3QG, Hertfordshire, U.K.; ¶ Department of Biochemistry and Systems Biology, Institute of Systems, Molecular and Integrative Biology, University of Liverpool, Liverpool L69 7ZB, U.K.

## Abstract

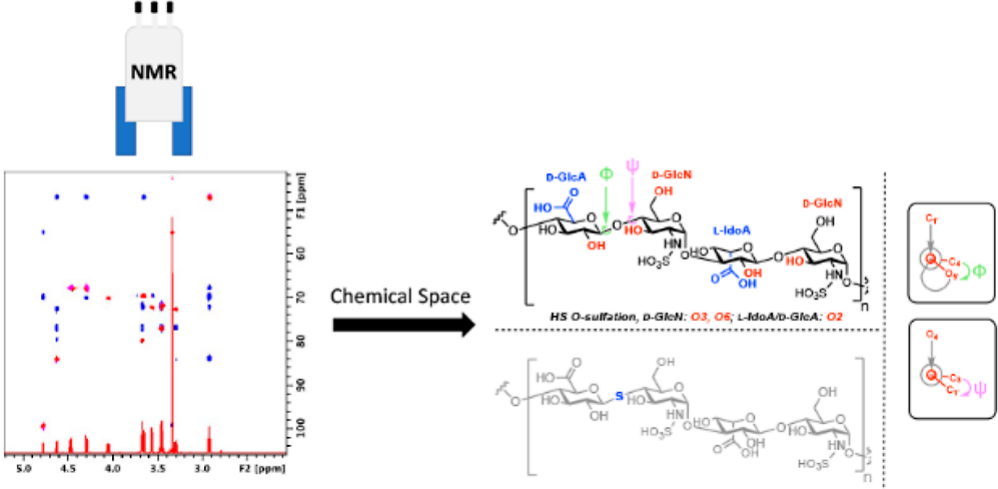

Heparan sulfate (HS), a sulfated linear carbohydrate that decorates the cell surface and extracellular matrix, is ubiquitously distributed throughout the animal kingdom and represents a key regulator of biological processes and a largely untapped reservoir of potential therapeutic targets. The temporal and spatial variations in the HS structure underpin the concept of “heparanome” and a complex network of HS binding proteins. However, despite its widespread biological roles, the determination of direct structure-to-function correlations is impaired by HS chemical heterogeneity. Attempts to correlate substitution patterns (mostly at the level of sulfation) with a given biological activity have been made. Nonetheless, these do not generally consider higher-level conformational effects at the carbohydrate level. Here, the use of NMR chemical shift analysis, NOEs, and spin–spin coupling constants sheds new light on how different sulfation patterns affect the polysaccharide backbone geometry. Furthermore, the substitution of native *O*-glycosidic linkages to hydrolytically more stable *S*-glycosidic forms leads to observable conformational changes in model saccharides, suggesting that alternative chemical spaces can be accessed and explored using such mimetics. Employing a series of systematically modified heparin oligosaccharides (as a proxy for HS) and chemically synthesized *O*- and *S*-glycoside analogues, the chemical space occupied by such compounds is explored and described.

## Introduction

**Figure 1:**
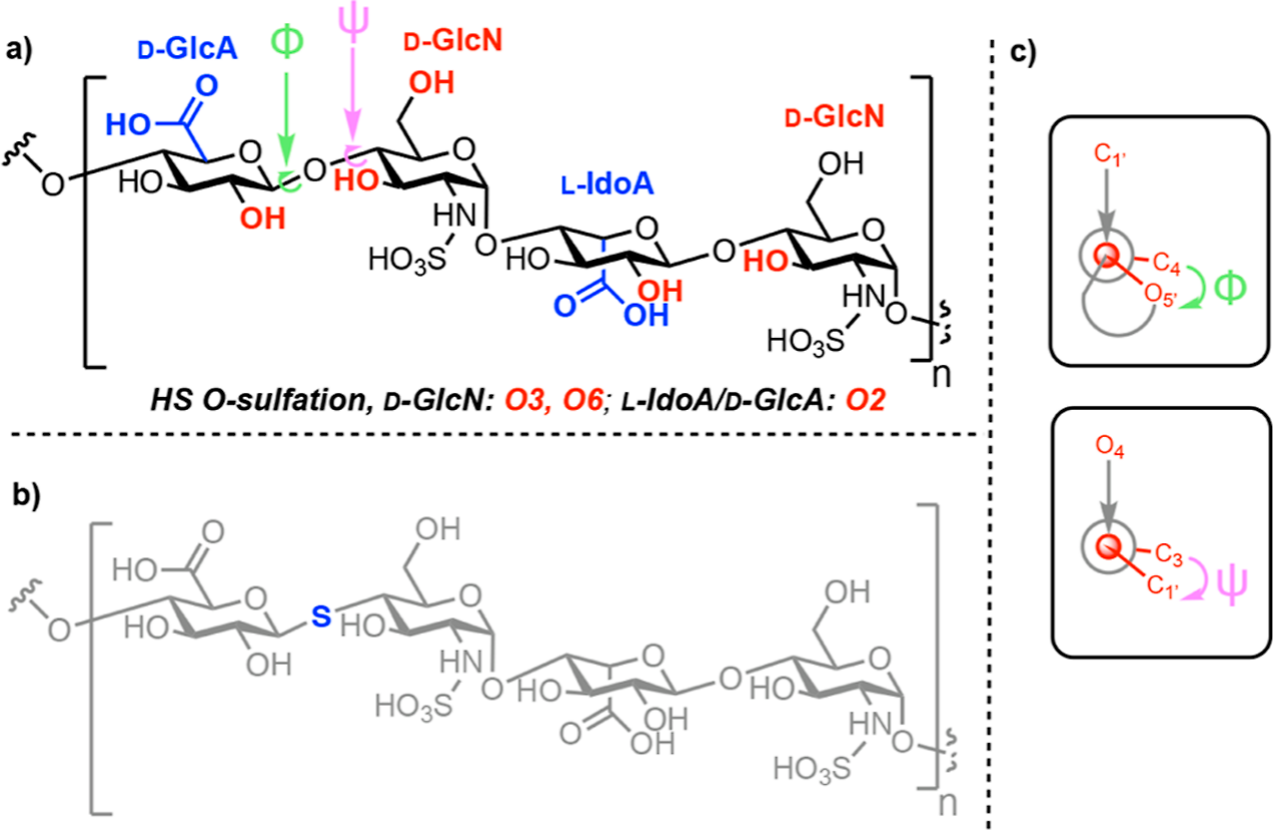
(a) Example HS tetrasaccharide with complete *N*-sulfation and illustrating uronate C5 epimers alongside points for *O*-sulfation; free acid form shown; (b) replacement of glycosidic oxygen with sulfur to create an *S*-glycoside analogue; and (c) interglycosidic torsion angles represented by Newman projections: Phi (Φ) is the O5′–C1′–O4–C4 angle and Psi (ψ) is the C1′–O4–C4–C3 angle, and O4 is the linkage oxygen.

The cell surface is decorated with complex carbohydrates, which form a dense and structurally diverse layer, known as the glycocalyx. Among these carbohydrates, the glycosaminoglycan heparan sulfate (HS) is composed of a backbone consisting of β- (1,4)-linked d-glucuronic acid (GlcA) and α- (1,4)-linked *N*-acetyl-d-glucosamine (GlcNAc) residues. This repeating disaccharide is further modified in regional blocks, whereby *N*-acetylglucosamine is *N*-sulfated instead, GlcA undergoes epimerization at C5 to l-iduronic acid (IdoA), and *O*-sulfate groups are added at several positions^1,2^ (Figure 1a).

During the biosynthesis of HS, these structural modifications do not go to completion at all sites within the polysaccharide, giving rise to structural heterogeneity within the final HS chain.^3,4^ Consequently, HS can bind and regulate countless proteins of biological significance^2,5−7^ and represents an untapped reservoir of therapeutic potential.^8^ As a result of this biological ubiquity, studies to probe and understand HS structure-to-function effects with molecular precision are imperative. The most famous example in this context is the heparin/antithrombin interaction, but recent studies have unveiled new molecular fingerprints underpinning the biological function.^9^

Access to, and analysis of, structurally homogeneous HS oligosaccharide sequences has developed significantly in recent years,^10−13^ and the glycan toolbox of materials now available to deconvolute HS structural complexity is impressive. Such a toolbox, composed of chemically modified and synthetic HS saccharides, can be used to inform the HS structure-to-function effects at a molecular level.^14,15^ Within this context, unnatural sequences such as those obtained from the replacement of glycosidic linkage oxygen with sulfur (*S*-glycosides, Figure 1b) offer the exciting possibility of studying unique conformational preferences about the thioglycosidic and aglyconic bonds.^16−18^ Subsequent comparison to native sequences will improve our understanding and capability to perturb HS structure-to-function relationships as well as access the untapped carbohydrate chemical space.

Herein, to deconvolute and inform future HS biological structure-to-function studies, we have produced and utilized a series of systematically modified heparin oligosaccharides (as a proxy for HS), alongside *O*- and *S*-linked HS model disaccharides. NMR analysis is used to dissect the global and local conformational effects for distinct substituent/heteroatom patterns.

## Materials and Methods

Heparin (UFH) from porcine mucosa was obtained from Bioiberica S.A. (Barcelona, Catalunya, Spain), while chemically modified heparin 6OH derivatives were prepared as described in ref (19). Briefly, *N*- and 6-*O*-desulfation was performed by heating heparin (200 mg, pyridinium salt) in a solution of DMSO containing 10% MeOH (25 mL) at 75 °C for 2 h. Re-*N*-sulfation was carried out by incubating the product of the latter reaction with the trimethyl-amine sulfur trioxide complex in a saturated sodium bicarbonate solution at 55 °C for 6 h. Oligosaccharides were isolated from heparinase digests of the polysaccharides separated on a Bio-Gel P-10 column (2.6 × 100 cm) at 0.5 mL/min in 0.25 M ammonium chloride previously calibrated with size-defined heparin oligosaccharides (Iduron, UK). Fractions (5 mL) were collected, and each peak was pooled separately, then desalted (HiPrep 26/10 Desalting column, GE Life Sciences), and freeze-dried. The peaks (Figure S1), corresponding to the size-defined heparin oligosaccharides, were detected by UV absorbance at 232 nm and had their structure confirmed by NMR (Figure S2).

### Synthesis of *O*- and *S*-HS Saccharide Analogues

**Scheme 1:**
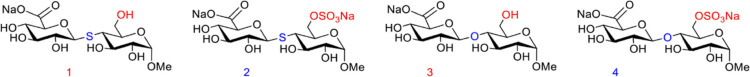
Chemical Structures of *O*- and *S*-HS Saccharide Analogues

*O*- and *S*-Disaccharides **1–4** were prepared as previously described.^16,20^ Briefly, *S*-disaccharides **1** and **2** were obtained by reacting (S_N_2) a suitable protected thiol derivative of GlcA (synthesized in four steps from d-glucurono-3,6-lactone) with a 4-triflate galactopyranoside precursor, followed by selective sulfation (for 2) and deprotection. *O*-Disaccharides **3** and **4** were prepared by glycosylation between a GlcA trichloroacetimidate donor and a 4-OH glucopyranoside derivative, followed by sulfation (for **4**) and deprotection. Scheme 1 depicts the structures of **1–4**.

### Nuclear Magnetic Resonance

Synthetic *O*- and *S*-HS saccharide analogues NMR experiments were performed at 300 K using an 800 MHz Bruker AVANCE III + spectrometer fitted with a CryoProbe. Unfractionated heparin, heparin oligosaccharides, and their chemically modified derivative NMR experiments were performed at 298, 310, or 343 K using a 500 MHz Bruker AVANCE NEO spectrometer fitted with a TXI probe. In addition to one-dimensional spectra, both homonuclear and heteronuclear two-dimensional spectra were collected. Total correlation spectroscopy (TOCSY, *dipsi2gpph19*) spectra were measured with a mixing time of 120–180 ms, while the mixing times for Nuclear Overhauser Effect Spectroscopy (NOESY, *selnogpz.2*) spectra varied between 120 and 300 ms. Heteronuclear Single Quantum Coherence (^1^H/^13^C HSQC, *hsqcedetgpsp.3*) spectra were collected using 32 scans, 2048 × 256 complex data points, spectral width in F1 5 KHz, in F2 11 KHz, and one-bond *J*_CH_ = 145 Hz. All J-resolved heteronuclear multiple bond correlation (HMBC) spectra, Bruker pulse program *hmbcetgpl3nd*, were collected at 800 MHz with the *J*_CH_ long-range value (cnst13) set to 3–8 Hz. The spectra were processed and assigned, and integration was performed using a Bruker TopSpin 4.1.4. Samples were prepared in deuterium oxide (>99.92%, Apollo Scientific) packed under argon to mitigate interferences arising from paramagnetic oxygen.

## Results and Discussion

### Distinct Sulfation Patterns Lead to Distant Conformational Effects in HS Oligosaccharides

Conventional NMR nuclei studies (mostly ^1^H and ^13^C) have been extensively used to provide details around conformational features of heparin/HS ligands.^21^ These studies have used natural, semisynthetic and fully synthetic materials with the same overall goal to extract structural information that can be correlated with biological function. NMR chemical shift analysis of HS polysaccharides has determined the extent to which different sulfation patterns (C2 of IdoA and C2 or C6 of GlcN) report on conformational changes throughout the polymer. For example, it was observed that sulfation at IdoA-C2 produces global conformation changes across the polymer backbone, whereas sulfation at C6 produces discrete changes, localized to the GlcN residue.^22^

**Figure 2:**
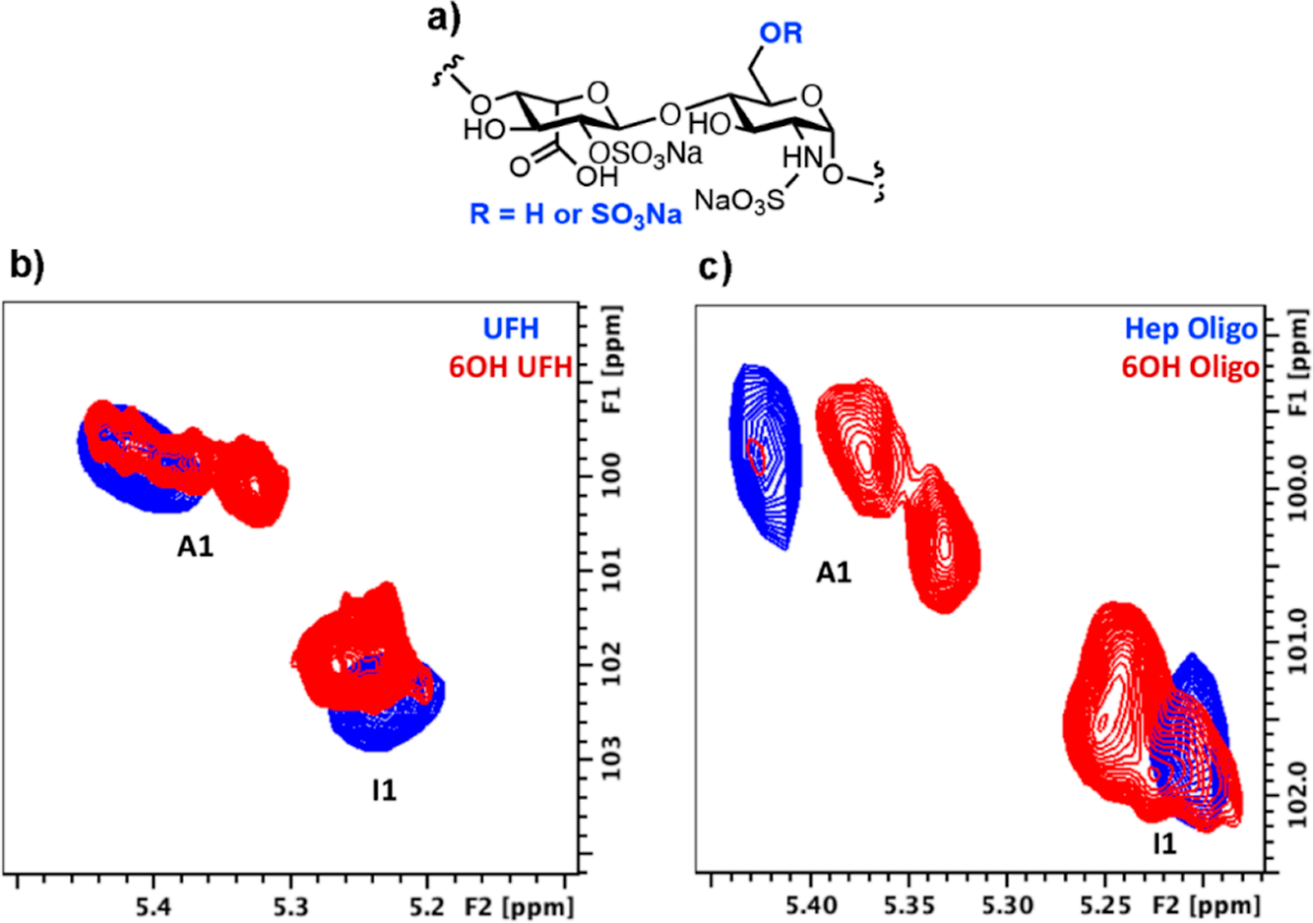
(a) General HS sequence structure for UFH and Hep oligos examined, (b) HSQC NMR spectra for anomeric regions of UFH and its 6OH derivative, IdoA (I) and GlcN (A1) residues, and (c) HSQC NMR spectra for anomeric regions of Dp12 and its 6OH derivative, IdoA (I) and GlcN (A) residues. 1 denotes H1, anomeric position.

Here, HS oligosaccharides were produced using selective desulfation of heparin.^19^ These materials were analyzed using NMR to see whether the data obtained for these simpler, shorter oligosaccharides was comparable to that previously observed at the polymer level. The HSQC NMR spectra for this series of HS oligosaccharides are shown in Figure 2. Accordingly, anomeric chemical shift data are tabulated in Table 1. As expected, and agreeing with previously published results,^22^ minor changes were observed in anomeric position (both IdoA and GlcN) ^1^H/^13^C chemical shifts when unfractionated heparin (UFH) was selectively desulfated at C6 (6OH UFH) (Figure 2a). Equally, the values observed for a heparin decasaccharide (Hep Oligo) and its 6OH variant (6OH Oligo, Figure 2b) presented minor changes again, the most significant chemical shift change occurring at GlcN. Furthermore, as for UFH, desulfation at C6 led to little/no changes at C4 (Figure S3); chemical shifts, remaining at around δ = 79 ppm, imply that 6-*O*-sulfation had little influence on the backbone and overall saccharide conformation, as judged by chemical shift changes.

**Table 1:** Key ^1^H/^13^C Anomeric Chemical Shift Data

	A1 (^1^H/^13^C ppm)	I1 (^1^H/^13^C ppm)
**UFH**	5.4/99.7	5.22/102.3
**6OH UFH**	5.35/99.7	5.23/101.9
**Hep Oligo**	5.42/99.7	5.21/101.6
**6OH Oligo**	5.35/100	5.23/101.6

### Probing Iduronate Flexibility Using NOEs Within Model HS Oligosaccharides

One of the key monosaccharide residues within HS, which bestows it with micro- and internal flexibility, is α-l-iduronic acid. Functionally, changes to the iduronate ring conformation seem to accommodate protein binding and lie at the heart of the prototypical interaction between heparin and AT.^23^ It is interesting to note, however, that the conformational properties of this residue, which involve fluidity through ^4^C_1_, ^1^C_4_, and ^2^S_0_ conformers, do not always lead to changes in overall chain orientation.^24^ Regardless, observations using model oligosaccharides may differ from those of polysaccharides. Building on the chemical shift analysis presented above, and since changes to the ring conformation alter the distance between vicinal hydrogen atoms, proton–proton NOEs were next used to probe iduronate ring conformation changes, correlating with distinct sulfation patterns of HS oligosaccharides. For this, selective irradiation of I5 was used, given its sensitivity to α-l-iduronic acid conformation and negligible overlap with signals from other protons (Figure SII).

**Figure 3:**
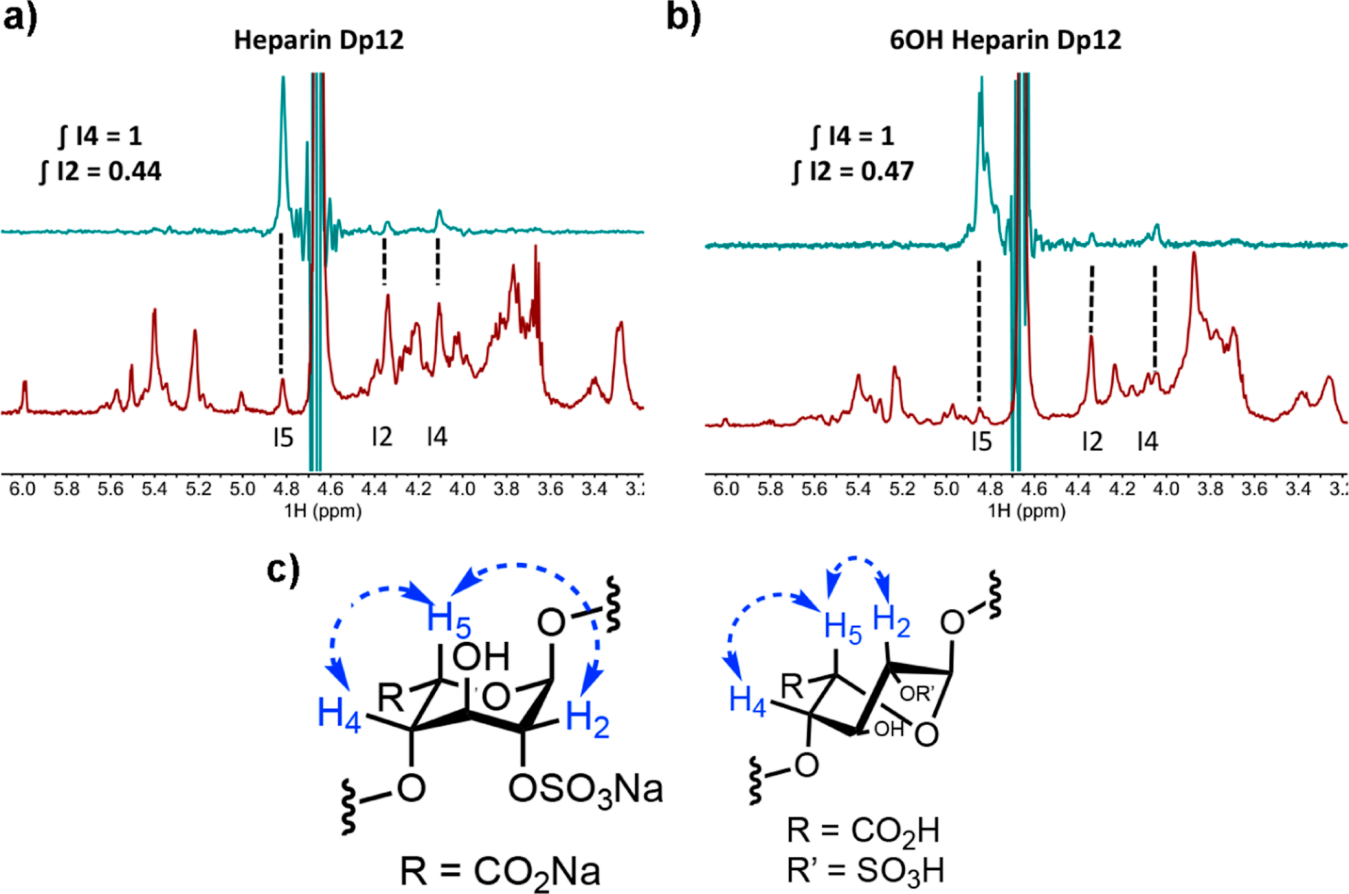
(a) Bottom, ^1^H NMR spectrum for heparin Dp12; top, selective 1D NOESY (irradiation of I5) with positions 5, 2, and 4 assigned; (b) bottom, ^1^H NMR spectrum for 6OH Dp12; top, selective 1D NOESY (irradiation of I5) with positions 5, 2, and 4 assigned; and (c) conformers ^1^C_4_ and ^2^S_0_ for IdoA2S residues which indicate distances between H2 and H5, and H4 and H5, displayed with dotted arrows.

Figure 3 shows selective 1D NOESY experiments for a series of chemically modified dodecasaccharides [degree of polymerization (Dp)12s]. The distribution of conformers was assessed by ^1^H–^1^H NOE analysis, which is particularly useful for the detection of the ^2^S_0_ conformer. In this conformer, the atomic distance between H2 and H5 (2.6 Å) compared with the ^1^C_4_ conformer (4.0 Å) can be easily extracted and inferred by measuring proton–proton NOEs: the shorter the distance, the stronger the NOEs,^23^ and any changes in equilibria between these would inevitably lead to changes in proton–proton NOE ratios. Sulfation at C6 of GlcN (Figure 3a,b) produced minor effects in the NOEs between I5 to I4 and I2 (judged by cross-peak integration), implying, once again, that such a modification position has a more localized effect in terms of saccharide conformation, which is confined around the GlcN residue. It is important to emphasize that this observation refers to the average conformation of all IdoA2S residues within a given chain since the resolution of NMR does not generally enable the differentiation between distinct residues positioned throughout the oligosaccharide. Furthermore, the dynamics of such conformer distributions is rather fast and, again, NMR does not provide a real-time picture of these events, rather, it provides the average over time.

### Effect of C6 Sulfation within *O*- and *S*-Disaccharide Analogues of HS

**Figure 4:**
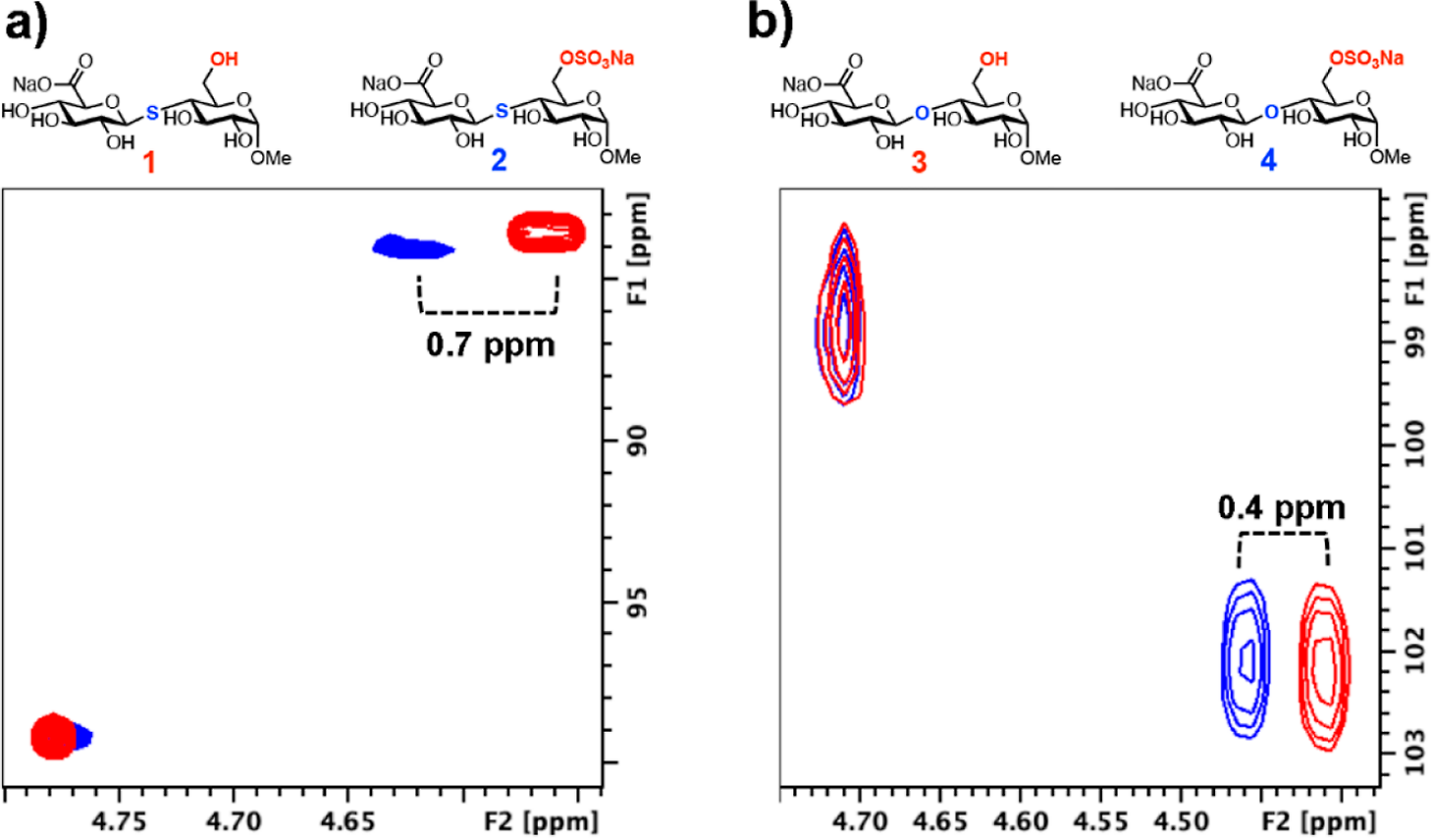
(a) HSQC NMR spectrum showing the effect of the 6-*O*-sulfation pattern upon *S*-glycosides and (b) HSQC NMR spectrum showing the effect of 6-*O*-sulfation pattern *O*-glycosides; black cross-peak = 6-OH compound and red cross-peak = 6-OSO_3_Na compound.

Utilizing *O*- and *S*-linked HS disaccharide analogues, bearing Glc in place of GlcN,^25^ the impact of C6 sulfation in shorter sequences was also investigated by NMR. Sulfation at C6 in *S*-glycoside **2** led to a significant change in ^1^H chemical shift of the adjacent uronic acid (0.7 ppm downfield change) when compared to the shift observed for its *O*-glycoside counterpart **4** (0.4 ppm) (Figure 4b). A small ^13^C chemical shift change at C1 (83.5 ppm for **1** to 84.05 ppm for **2**) of sulfated *S*-glycoside uronate was also observed. These results suggest that instead of a localized change in the chemical environment in the case of *O*-glycoside **4**, sulfation at C6 of *S*-glycoside **2** led to global changes in the overall saccharide chemical environment, as adjudged by the uronate chemical shift changes. These differences can be associated with changes in linkage geometry and changes to the uronate conformer (as evidenced by *J* coupling values, *vide infra*). Such an effect could be rationalized by the fact that sulfur is less electronegative than oxygen, increasing the shielded environment around the linkage, enabling the C6 sulfate to impart long-range conformational changes elsewhere in the chain.

### Spin–Spin Couplings Suggest Distinct Uronate Conformer Equilibria and Interglycosidic Dihedral Angles for *O*- and *S*-Linked HS Disaccharide Analogues

**Figure 5:**
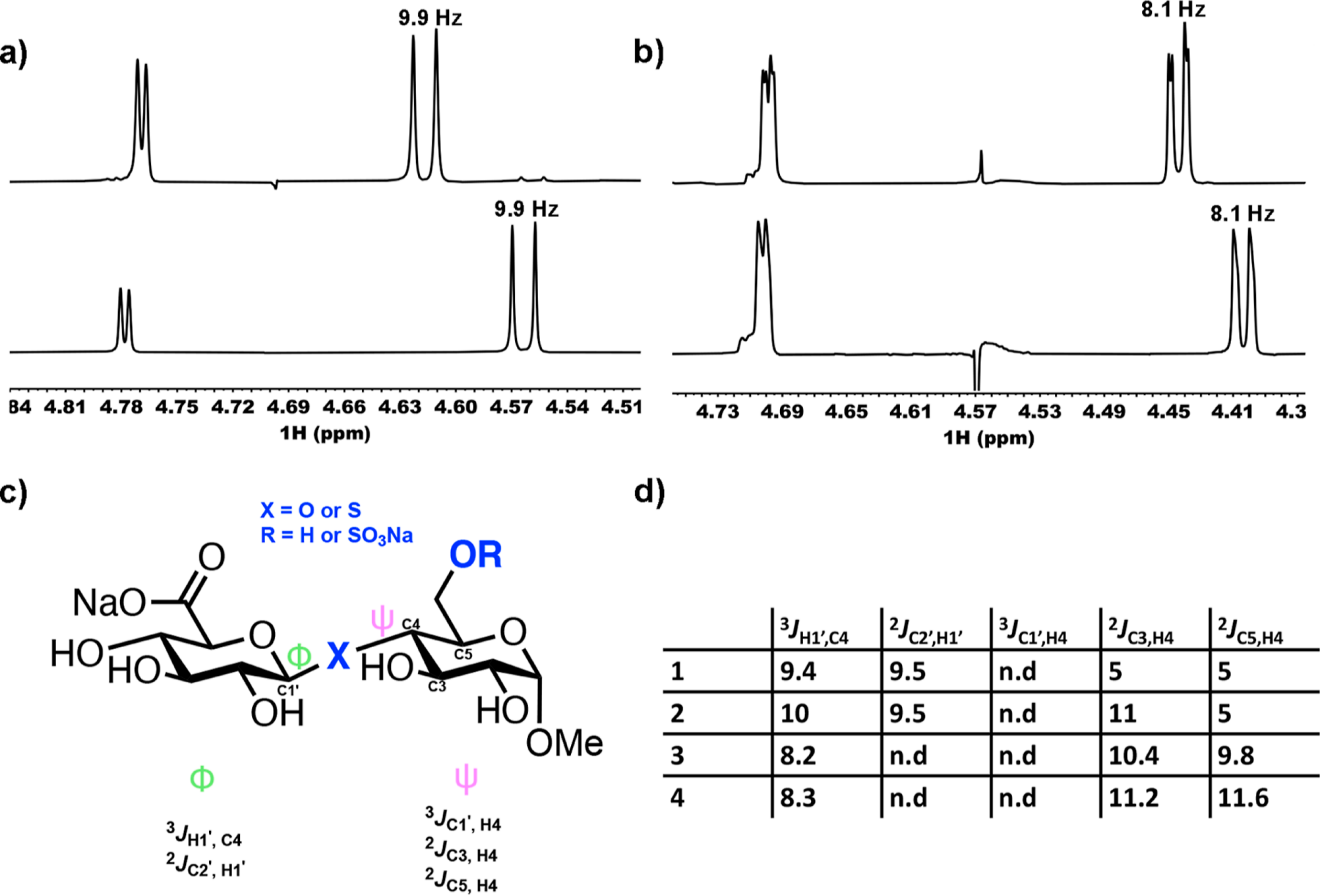
(a) Anomeric region of ^1^H NMR spectra from 6-OH and 6-OSO_3_Na *S*-glycosides **1** and **2** (bottom to top), (b) anomeric region of ^1^H NMR spectra from 6-OH and 6-OSO_3_Na *O*-glycosides **3** and **4** (bottom to top), (c) scheme showing redundant *J*-couplings across the β (1 → 4) *O*- and *S*-glycosidic linkages, and (d) redundant *J*-couplings across the β (1 → 4) *O*- and *S*-glycosidic linkages values in Hz. n.d, not detectable.

The conformational characteristics of glycans are, in part, influenced by the flexibility of their saccharide conformer equilibria and glycosidic linkages. ^1^H NMR analysis provides not only information around the constituent monosaccharides, based on the chemical shift, but also information around the dihedral angle between two coupled protons,^26^ for example, the non-equivalent protons on C1 and C2. Figure 5a,c shows the spin–spin coupling constants (^3^*J*_H1,H2_ of GlcA) for both *O*- and *S*-glycoside analogues of HS, **1–4**. There were no observable differences resulting from sulfation at C6 for either **3** or **4** (^3^*J*_H1,H2_ = 8.1 Hz for **3** to **4** and 9.9 Hz for **1** to **2**). However, switching from an *O*-glycosidic linkage to an *S*-glycoside led to an increase of 1.8 Hz (from 8.1 to 9.9 Hz) for this ^3^*J* coupling, suggesting a change in the solution-phase glucuronic acid conformation, certainly about the C1–C2 bond.

Long-range heteronuclear coupling constant analysis using *J*-resolved NMR experiments were next employed to ascertain the effects of C6 sulfation and conversion of *O*- to *S*-glycosidic linkages upon glycosidic torsion angles. Dihedral angles across such linkages require four atoms linked by three consecutive bonds and represent the rotation angle between two planes around the middle bond (the saccharide units). Such angles can range from −180 to 180°, depending on the direction of rotation, are named phi (ϕ) and psi (ψ), and are illustrated in Figure 1c.^27^

As some of these constants are extremally small and at times undetectable (when dihedral angles are around 90°), resolution was sufficient at 800 MHz to enable measurement of only some of these. Yet, measuring Redundant *J*-couplings,^28^ which have been used to model conformational populations of *O*-glycosidic linkages, facilitated the further investigation into the effects of C6 sulfation and *O*- to *S*-glycosidic conversion upon glycosidic torsion angles (Figure 5c). Again, the *O*- to *S*-replacement led to changes in these coupling constants (Figure 5d), suggesting differences in linkage behavior. *O*-glycan sulfation at C6 did not induce changes in ^3^*J*_H1’,C4_ values (Figure 5d, 8.2 Hz for **3** and 8.3 Hz for **4**), whereas a difference of 0.6 Hz was observed due to C6 sulfation in thioglycoside **2** (Figure 5d). Furthermore, there were significant changes to ^2^*J*_C3,H4_ in **1** and **2**, which further suggest interglycosidic dihedral angle variations. Such changes may be explained by the shielding effect of sulfur *versus* oxygen (mentioned above) and longer C–S thioether bonds. Together, these results demonstrate that *S*-glycosides display conformational states that are unique from their natural *O*-glycosides.

## Conclusions

The overall conformation of *O*- and *S*-glycoside analogues of HS is dependent on the substitution pattern within such molecules and their glycosidic linkage type. This observation agrees with previously described effects using systematically modified heparin polymers as a proxy for HS. Much of this also underpins many structure-to-function relationships and reinforces the idea that saccharides of similar backbones presenting different substitution patterns will display unique molecular architecture affecting their geometry, conformation, flexibility, and thus biological effect.

The conformational changes presented by *S*-glycoside analogues differ from those observed for their *O*-linked counterparts, highlighting that this heteroatom substitution enables access to previously untapped chemical space, which could be exploited as a powerful tool in investigating glycosaminoglycan–protein interactions and possibly inform the design of druggable glycan-based candidates.

## Supporting Information Available

Biogel P10 oligosaccharide fractionation; HSQC spectra for heparin and 6OH heparin oligosaccharide; HSQC spectra showing the C4 region of 6S (blue) and 6OH (red) heparin derivatives; and *J*-resolved HMBC spectra for *S*- and *O*-glycosides (PDF)
