# Supplementary material for: Using NMR to Dissect the Chemical Space and O-Sulfation Effects within the O- and S-Glycoside Analogues of Heparan Sulfate
Source: ACS Omega. 2022 Jul 8;7(28):24461–7. doi: 10.1021/acsomega.2c02070 (PMC9301708; doi:10.1021/acsomega.2c02070)
Supplement: Supplementary file 1 — ao2c02070_si_001.pdf [file ao2c02070_si_001.pdf]

Using NMR to dissect the chemical space and *O*-sulfation effects within *O*- and *S*-glycoside analogues of heparan sulfate

Maria C.Z. Meneghetti[a#], Lucy Naughton[b,c#], Conor O'Shea[d,c#], Dindet S.-E. Koffi Teki[e], Vincent Chagnault[e], Helena B. Nader[a], Timothy R. Rudd[f,g], Edwin A. Yates[g], José Kovensky[e,*], Gavin J. Miller[d,c*] & Marcelo A. Lima[b,c*@]

[a] Departamento de Bioquímica, Instituto de Farmacologia e Biologia Molecular, Escola Paulista de Medicina, Universidade Federal de São Paulo, Rua Três de Maio, 100, São Paulo, SP 04044-020, Brazil

[b] School of Life Sciences, Keele University, Keele, Staffordshire, ST55BG, UK

[c] Centre for Glycosciences, Keele University, Keele, Staffordshire, ST55BG, UK

[d] Lennard-Jones Laboratories, School of Chemical and Physical Sciences, Keele University, Keele, Staffordshire, ST55BG, UK

[e] Laboratoire de Glycochimie, des Antimicrobiens et des Agroressources (LG2A), UMR 7378 CNRS, Université de Picardie Jules Verne, 33 rue Saint Leu, F-80039 Amiens Cedex, France

[f] National Institute for Biological Standards and Control (NIBSC), Blanche Lane, South Mimms, Potters Bar, Hertfordshire, UK

[g] Department of Biochemistry and Systems Biology, Institute of Systems, Molecular and Integrative Biology, University of Liverpool, Liverpool, UK

# These authors are joint first authors

* Senior authors

[@] Correspondence: Marcelo A. Lima, Centre for Glycosciences, Keele University, Keele, Staffordshire, ST55BG, UK, m.andrade.de.lima@keele.ac.uk

The current address for TRR is Analytical and Biological Sciences, Research & Development, Science, Research and Innovation, Medicines & Healthcare products Regulatory Agency, Blanche Lane, South Mimms, Hertfordshire, EN6 3QG, UK

Supplementary figures

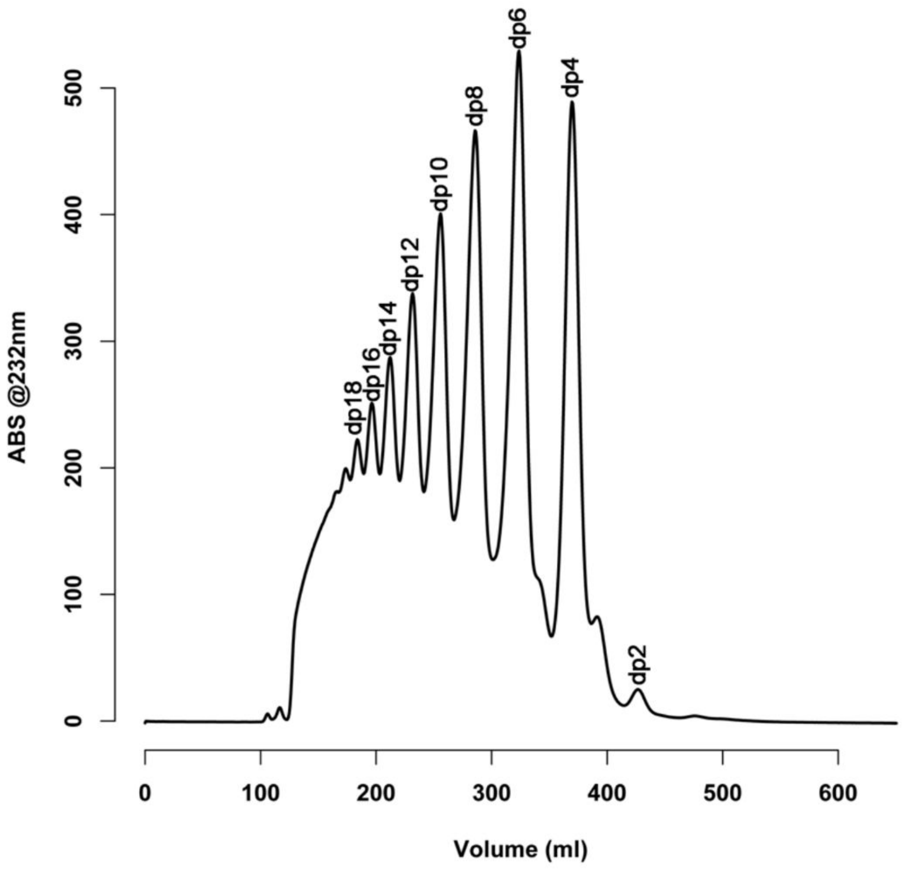

Figure S1. Biogel P10 oligosaccharide fractionation.

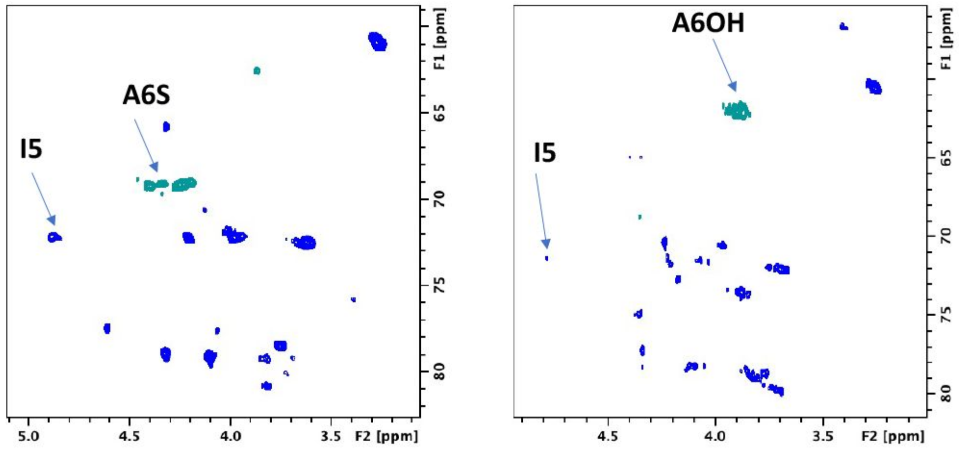

Figure S2. HSQC spectra for heparin and 6OH heparin oligosaccharide.

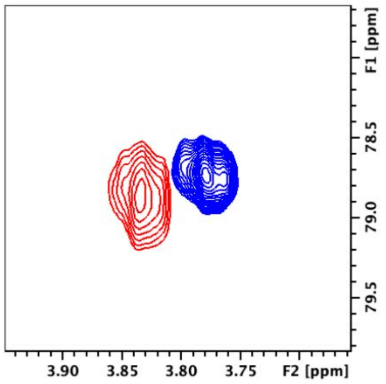

Figure S3. HSQC spectra showing the C4 region of 6S (blue) and 6OH (red) heparin derivatives.

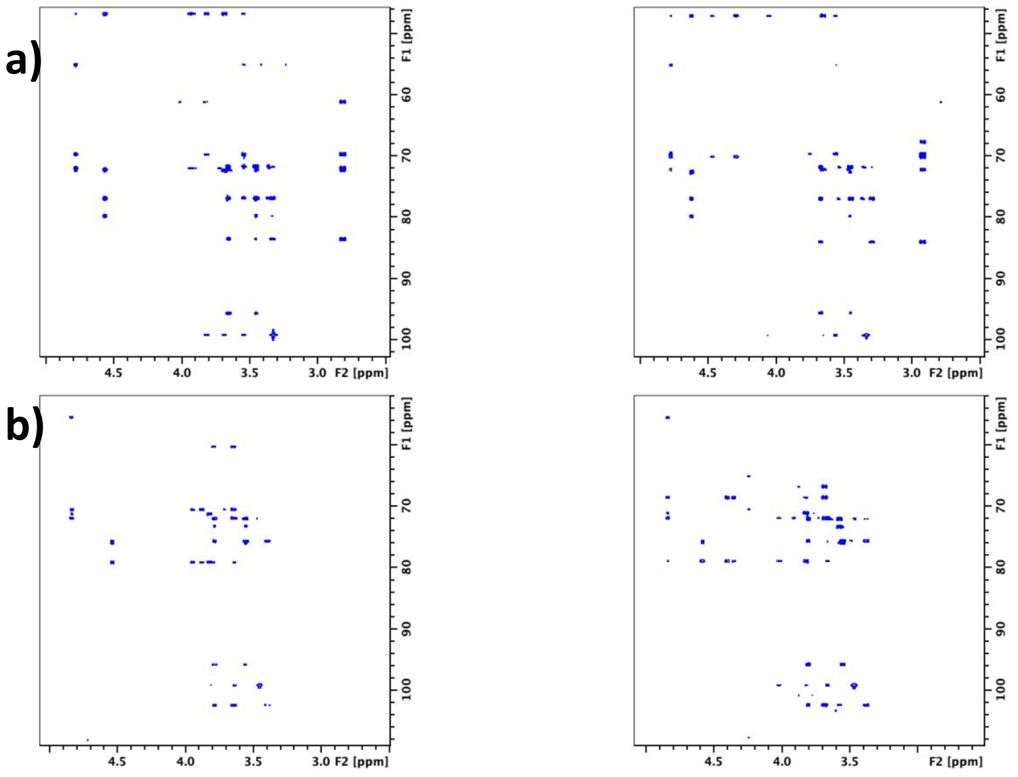

Figure S4. a) J-resolved HMBC spectra for 6-OH and 6-OSO3H S-glycosides 1 (left) & 2 (right) **b)** J-resolved HBMC spectra for 6-OH and 6-OSO3H O-glycosides 3 (left) & 4(right).
